# Low‐Magnitude High‐Frequency Vibration Attenuates Sarcopenia by Modulating Mitochondrial Quality Control via Inhibiting miR‐378

**DOI:** 10.1002/jcsm.13740

**Published:** 2025-02-19

**Authors:** Yu‐Feng Long, Can Cui, Qianjin Wang, Zhen Xu, Simon Kwoon‐Ho Chow, Ning Zhang, Ronald Man Yeung Wong, Elvis Chun‐Sing Chui, Rebecca Schoenmehl, Christoph Brochhausen, Clinton Rubin, Gang Li, Ling Qin, Da‐Zhi Yang, Wing‐Hoi Cheung

**Affiliations:** ^1^ Musculoskeletal Research Laboratory, Department of Orthopaedics and Traumatology The Chinese University of Hong Kong Hong Kong China; ^2^ Li Ka Shing Institute of Health Sciences The Chinese University of Hong Kong Hong Kong SAR China; ^3^ Department of Spine Surgery The 6th Affiliated Hospital of Shenzhen University Health Science Center Shenzhen China; ^4^ Translational Medicine R&D Center, Institute of Biomedical and Health Engineering, Shenzhen Institute of Advanced Technology Chinese Academy of Sciences Shenzhen China; ^5^ Department of Orthopaedic Surgery Stanford University Stanford California USA; ^6^ Institute of Pathology, University Hospital Mannheim University of Heidelberg Germany; ^7^ Department of Biomedical Engineering Stony Brook University Stony Brook New York USA

**Keywords:** miR‐378, mitochondria, sarcopenia, skeletal muscle, vibration

## Abstract

**Background:**

Sarcopenia, the age‐related decline in muscle mass and muscle strength, significantly contributes to falls, diminished quality of life, and mortality. Although mitochondrial dysfunction is increasingly implicated in sarcopenia, the underlying mechanisms are not fully discovered. Low‐magnitude high‐frequency vibration (LMHFV), a recommended treatment by the Centers for Disease Control and Prevention (CDC) to reduce fall risk, remains poorly understood of the mechanism on improving skeletal muscle quality. This study aims to investigate whether mitochondrial dysfunction contributes to sarcopenia and evaluate whether LMHFV mitigates sarcopenia by improving mitochondrial homeostasis.

**Methods:**

The relationship between mitochondria dysfunction and sarcopenia using senescence accelerated mice prone 8 (SAMP8) model was investigated, assessing muscle and mitochondria. The effects of LMHFV on muscle and mitochondria were evaluated in SAMP8 mice during sarcopenia progression. The role of miR‐378 in muscle and mitochondrial homeostasis were evaluated in SAMP8 mice and transgenic over‐expressing miR‐378 mice (TG mice). The target gene of miR‐378 was investigated by dual‐luciferase reporter assay in C2C12 cells. Subsequently, we evaluated the effect of LMHFV on miR‐378 using both mouse models.

**Results:**

Reduction in muscle strength was observed from the ages of month 8 to 10 in SAMP8 mice (grip strength decreased 27.1%, *p* = 0.0263; twitch force decreased 29.1%, *p* = 0.0178; tetanic force decreased 29.9%, *p* = 0.011), as well as muscle atrophy (cross‐section area: 38.3%, *p* = 0.0121). Mitochondrial morphological deterioration was noticed from month 6 to 10. Mitochondrial homeostasis, including biogenesis, fusion, fission, mitophagy, and ATP production declined from month 6 to 10. Compared to control group at month 10, knocking down miR‐378 in SAMP8 mice mitigated sarcopenia (twitch force increased 44.3%, *p* = 0.0023; tetanic force increased 51.9%, *p* = 0.0005), improved mitochondrial morphologies (mitochondrial number increased 1.65‐fold, *p* = 0.0023; mitochondrial density increased 1.65‐fold, *p* = 0.0023; mitochondrial relative area increased 9.05‐fold, *p* = 0.0019) along with improved mitochondrial homeostasis. Over‐expressing miR‐378 in transgenic mice exacerbated muscle atrophy and mitochondrial deterioration significantly. The dual‐luciferase reporter assay in C2C12 cells revealed that miR‐378 inhibited PGC‐1α directivity. LMHFV was found to mitigate sarcopenia by modulating mitochondrial homeostasis, such as attenuating mitochondrial morphological deterioration and improving mitochondrial biogenesis through increasing PGC‐1α via inhibiting miR‐378 in skeletal muscle.

**Conclusions:**

Our findings indicate that mitochondrial biogenesis, fusion, fission, and mitophagy were compromised during progression of sarcopenia, with mitochondrial deterioration preceding the onset of sarcopenia symptoms. The study also demonstrated that LMHFV could attenuate sarcopenia by modulating mitochondrial quality control through inhibiting miR‐378, highlighting its therapeutic potential in the management of age‐related muscular degeneration.

## 1 Introduction

Sarcopenia is a hallmark of the ageing process and a syndrome characterized by progressive and generalized loss of skeletal muscle mass and muscle performance, accompanied by several adverse outcomes including frailty, poor quality of life, and even mortality [1, 2, 3]. Sarcopenia influences 10%–16% of the elderly worldwide, and with a growing elderly demographic, sarcopenia will become more common [4]. Therefore, it is urgent for us to have a better understanding of sarcopenia, which will be the basis for developing more effective treatments in the ageing society.

Mitochondria are crucial organelles providing energy for biological processes, with their dynamic equilibrium being pivotal in preserving their functionality. Mitochondrial biogenesis, fusion, fission, and mitophagy are integral in maintaining this balance [5]. Although recent studies indicated that sarcopenia was associated with mitochondrial dysfunction [6], it was uncertain if sarcopenia was the consequence or cause of mitochondrial dysfunction [7] and the specific mechanism remain partially unclear [2, 8]. MicroRNAs, small single‐stranded RNAs of ~23 nucleotides, are central to the post‐transcriptional regulation of gene expression and protein levels [9]. Certain miRNAs are abundant in skeletal muscles and are instrumental in muscle development and function regulation [9, 10]. They also influence mitochondrial function by interacting with mitochondrial DNA [11]. For instance, miR‐378 has been linked to muscle atrophy [12] and impacts on mitochondrial performance [13]. Therefore, exploring the role of miR‐378 could unveil new insights into sarcopenia associated with ageing and lead to innovative treatments.

It is well‐established that physical exercise can influence many changes in muscle during ageing, which is essential to healthy ageing [14]. Furthermore, numerous studies have shown that physical exercise is an effective treatment to mitigate sarcopenia [15, 16, 17]. It was indicated that exercise enhanced muscle performance by regulating mitochondria [18, 19, 20] through modulating mitochondrial biogenesis [21], fusion and fission, or mitophagy [6]. Additionally, our previous review suggested that both endurance and resistance exercises could improve muscle mass and strength by modulating skeletal muscle mitochondria via regulating microRNAs [10]. Regular physical exercise is the clinically recommended gold standard for treating sarcopenia among the old population [22], while some elderly, especially those physically unfit, showed reluctance to exercise regularly and effectively due to a fear of falling, lack of motivation, or a general dislike of exercise [23].

Low‐magnitude high‐frequency vibration (LMHFV) treatment is a kind of intriguing intervention with a specific device that provides an oscillation at 35 Hz, 0.3 g (peak‐to‐peak magnitude) with displacement of < 0.1 mm to the whole body. Our previous studies illustrated that LMHFV can improve muscle strength and balancing abilities in elderly, as well as attenuating sarcopenia without adverse effects in animals [24]. Furthermore, the CDC has recommended using LMHFV as a preventive measure against falls in elderly [25]. Importantly, comparing with daily exercise, LMHFV is a more acceptable approach with lower falling risk for elderly people to get effective treatment by simply standing on the platform [25]. However, the link between LMHFV and mitochondria in skeletal muscle remains unknown. Therefore, investigating the mechanisms among LMHFV, mitochondria, and miRNAs may help us develop new therapies for skeletal muscle disorders, especially for those who are not fit to do regular exercise.

## 2 Methods

### 2.1 Animals

The study was conducted on male wild‐type C57BL/6 mice, miR‐378 over‐expressing transgenic mice, and senescence‐accelerated mouse P8 (SAMP8 mouse) acquired from the Laboratory Animal Service Center (LASEC) at the Chinese University of Hong Kong. The study was ethically approved by the Animal Experimentation Ethics Committee (AEEC), the Chinese university of Hong Kong (Ref. No. 21‐073‐HEA and 22‐265‐MIS). After anaesthesia, the functional and structural outcomes of skeletal muscle of mice were assessed at designated time points. The gastrocnemius (GA) was used for *ex‐vivo* muscle functional test and myofiber staining, the extensor digitorum longus (EDL) muscle for RNA extraction and real‐time quantitative PCR (RT‐qPCR) test; and the tibialis anterior (TA) muscle for protein extraction and western blot analysis. SAMP8 mice were randomly assigned to 3 groups: control (CTL), LMHFV treatment (VIB) and miR‐378 knockdown (KD). The transgenic mice used in this study were C57BL6 genetic background and miR‐378 was over expressed in the whole body [26]. C57BL/6 mice were included as a WT group, and transgenic mice were in the TG group. Tissue samples from the WT, TG, and T + V (TG mice with LMHFV treatment) groups were collected at month 8 for further experiments.

### 2.2 LMHFV Treatment

All mice from VIB and T + V groups were treated with LMHFV intervention when they were 6 months old. During the treatment, the mice were individually housed in a standard and compartmented bottomless cage on a custom‐designed vibration platform (V‐health Ltd, Hong Kong) (Figure S1a). The LMHFV treatment (35 Hz, 0.3 g; g = gravitational acceleration; 20 min/day, 5 days/week) was administered based on our previous studies [27, 28]. Additionally, TG mice receiving LMHFV treatment at month 6 formed the T + V group (Figure S1a). Meanwhile, mice in CTL or WT group were put on the platform without vibration as a sham treatment.

### 2.3 Knocking Down miR‐378

To knock‐down miR‐378 expression, the adeno‐associated virus 9 (AAV9) carrying the functional sequence was utilized in SAMP8 mice at 6 months of age in KD group. pAAV‐U6‐TuD (mmu‐miR‐378)‐CMV‐EGFP‐WPRE (OBiO Technology, China) was employed for multi‐point injections into the TA, EDL, and GA muscles in both legs, following manufacturer's protocols. The information of miR‐378 was given in Table S2.

### 2.4 AAV9 Multi‐Point Local Injections in Skeletal Muscle

Multi‐point local injections were performed under anaesthesia **in both legs** when the mice were 6 months old (Figure S1b), with each injection point having a concentration of 2.0E+10 v.g./mL. For the TA and EDL muscles, a 4‐point injection was conducted using 20 μL of AAV9 using micro syringe (10 μL volume, 35G needle, Hamilton, USA). Meanwhile, a total of 30 μL of AAV9 was injected into the GA muscle at four different points using micro syringe (Figure S1b). Additionally, the AAV9 with scrambled sequence were injected into these SAMP8 mice at 6 months in the KD‐NC group, which the volume, concentration and injection points were the same as KD group.

### 2.5 Grip Strength Measurement

The forelimb grip strength of mice was measured using a force gauge (Mark‐10 Corporation, Copiague, USA). Following our previous studies [24, 29], the tails of mice were gradually pulled until they released their forelimbs from the grid. Each mouse's grip strength was evaluated 3 times and averaged.

### 2.6 Ex‐Vivo Muscle Functional Test

The GA muscle was selected as the target muscle due to its essential roles in body movement and posture maintenance. The *ex‐vivo* muscle functional test followed our established protocol [24, 28, 29] (Figure S1c). Briefly, GA muscle was electronically stimulated (at 150 Hz for 300 ms) to get the maximum twitch force at an optimal length. Then, the twitch and tetanic forces were evaluated and analysed by the Dynamic Muscle Analysis system (DMA v5.3.2).

### 2.7 Histological Analysis

The gastrocnemius muscles were collected and cryo‐sectioned for H&E staining. Muscle samples sectioned at 10 μm thick (Cryostar NX70, Thermo Scientific, MA, USA) were mounted on silane‐coated glass slides [29, 30]. We used ImageJ software (NIH, Bethesda, USA) to measure the cross‐sectional area of each muscle fibre, then summed the individual CSAs and divided by the number of fibres measured. Additionally, transmission electronic microscopy (TEM) was employed to observe morphological changes in mitochondria within the gastrocnemius muscle. Mitochondria number, density, and relative area were further quantified using ImageJ software.

### 2.8 RNA Extraction and Real‐Time PCR

The EDL muscle, a fast‐twitch muscle, serves as an appropriate muscle for real‐time PCR, as demonstrated in our previous study [24]. The total RNA from the EDL muscle was harvested using RNAiso Plus reagent (TaKaRa, Japan), following the manufacturer's protocol. Reverse transcription (RT) reaction was performed using miRCURY LNA miRNA PCR Starter Kit (QIAGEN, USA). For real‐time quantitative PCR (RT‐qPCR), miRCURY LNA miRNA PCR Starter Kit (QIAGEN, USA) was employed, following the manufacturer's instructions. The real‐time PCR reaction was performed on the Life Technology QuantStudio 12K Flex qPCR (Thermo Fisher Scientific, USA), based on the manufacturer's instructions.

### 2.9 Cell Culture and Dual‐Luciferase Reporter Assay

The dual‐luciferase reporter assay was performed using C2C12 cells. In the NC group, miR‐378 with a scramble sequence was transfected. In the Mut + miR‐378 group, miR‐378 mimics with a mutated sequence alignment of PGC‐1α‐3’UTR was used, while miR‐378 mimics was applied in the WT + miR‐378 group. C2C12 myoblast cells were maintained in Dulbecco's modified minimum essential medium (DMEM) supplemented with 10% foetal bovine serum (Gibco, USA), 3.7 g/L sodium bicarbonate (Sigma Aldrich, USA) and 1% Penicillin–Streptomycin‐Neomycin (PSN) Antibiotic Mixture (Gibco, USA) at 37 °C in air with humidified atmosphere of 5% CO_2_. C2C12 cells were planted in a 48‐well plate and co‐transfected with miR‐378 and primGLO reporter plasmids (GenePharm, China) when the concentration of myoblast at 50%. Forty‐eight hours after transfection, according to the manufacturer's instructions, cells were lysed and luciferase activity was analysed with the Dual Luciferase Reporter Assay System (Promega, USA) on a luminometer (BioTek SynergyH1, USA).

### 2.10 Protein Extraction and Western Blot Analysis

For protein extraction, the TA muscle, a fast‐twitch muscle, was used. The cytoplasmic and mitochondrial proteins from the TA muscle were extracted following a modified protocol [31]. Both mitochondrial protein and cytoplasmic proteins were collected. Subsequently, Western blot analysis was performed according to our established protocol [29]. Primary antibodies and secondary antibodies used are shown in Table S1.

### 2.11 Statistical Analysis

All quantitative data were expressed as mean ± standard deviation. GraphPad Prism 8.02 (GraphPad Software, USA) was employed for statistical analysis. One‐way ANOVA followed by post‐hoc Tukey's test was used to compare different groups. Statistical significance was set at *p* < 0.05.

## 3 Results

### 3.1 Muscle Performance Decreased Accompanied by Increasing miR‐378 During Sarcopenia Progression

Our previous studies indicated that sarcopenia onset occurred at month 8 in SAMP8 mice model [24, 28]. In this study, we revealed dynamic alternations in grip strength, twitch force, and tetanic force over time. These parameters increased from month 3 to 8 but subsequently decreased from month 8 to 12 (Figure S2a). Interestingly, muscle performance showed no significant difference between month 10 and month 12 (Figure S2a).

**FIGURE 1:**
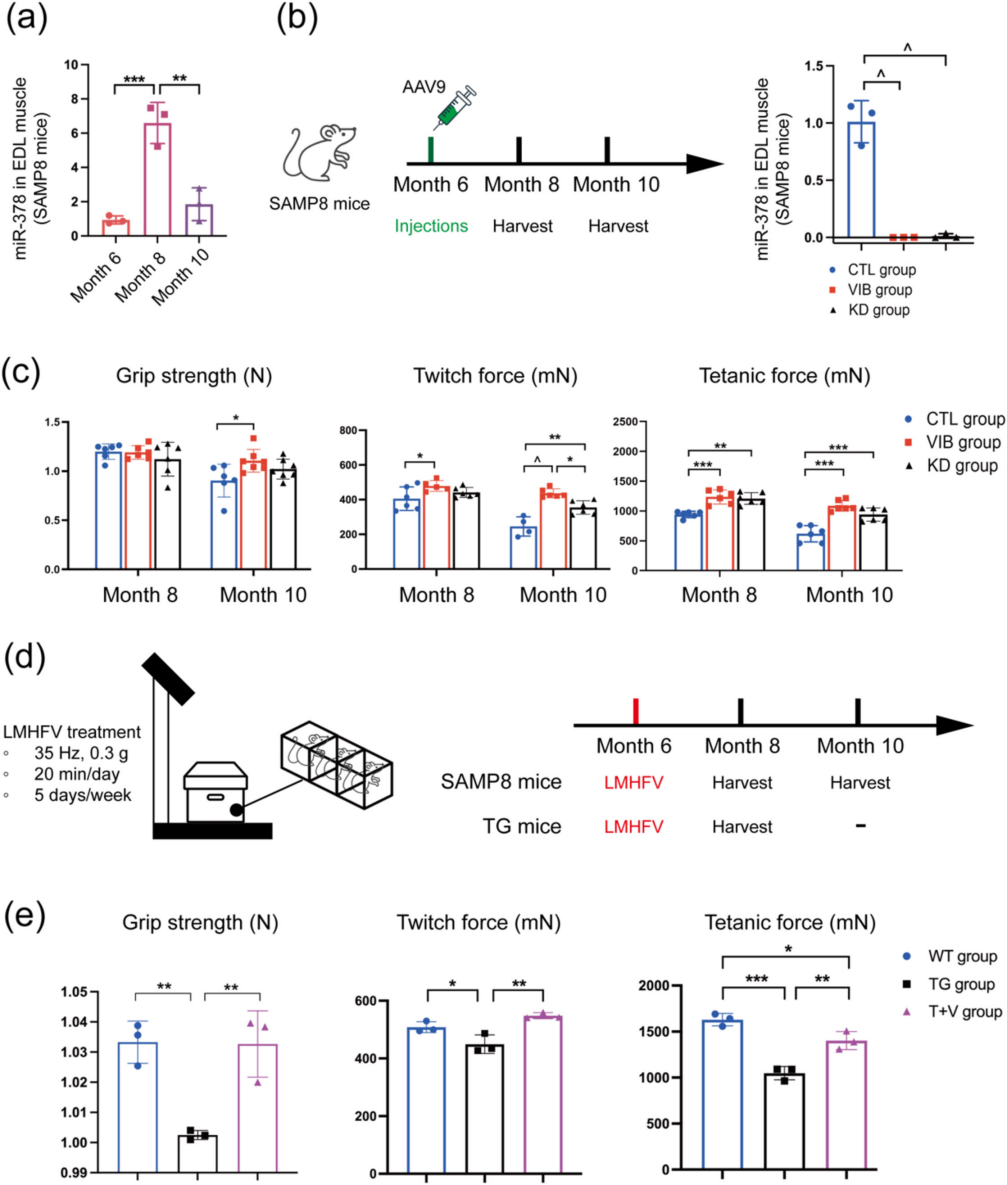
Skeletal muscle performance increased after LMHFV treatment or knockdown mir‐378. (a) The miR‐378 expression level in EDL muscle from SAMP8 mice. (b) Schematic diagram for knocking down miR‐378 and the miR‐378 expression level in EDL muscle from SAMP8 mice in different groups. (c) The grip strength, twitch force, and tetanic force of SAMP8 mice at month 8 and 10. (d) Schematic diagram for LMHFV treatment. (e) The grip strength, twitch force, and tetanic force of wild‐type mice and transgenic mice at month 8. Means ± SD are shown, *N* = 3, *p < 0.05, **p < 0.01, ***p < 0.007, ^*p* < 0.0001, one‐way ANOVA.

To investigate the effect of miR‐378 on progression of sarcopenia, we first evaluated the level of miR‐378 in a SAMP8 mice model. The results showed that miR‐378 increased from month 6 to 8 but dropped from month 8 to 10 (Figure 1a). Interestingly, we also found that miR‐378 increased from month 3 to 21 in EDL muscle from wide‐type mice (Figure S2b). Similarly, the data showed that miR‐378 in SOL muscles, which are slow‐twitch muscles, increased from month 3 to 21 (Figure S2b), indicating that the level of miR‐378 did not vary between different muscle types during sarcopenia progression.

### 3.2 MiR‐378 Damaged Muscle Performance During Sarcopenia Progression

To further investigate the effect of miR‐378 on muscle performance, miR‐378 was knocked down by performing AAV9 multi‐point local injections in GA, TA, and EDL muscles of 6‐month‐old SAMP8 mice (Figure 1b). The level of miR‐378 was significantly reduced in KD group at month 8 (Figure 1b) and AAV9 was efficiently transfected to the GA muscle at month 8 and the effect persisted to month 10 (Figure S2c).

Subsequently, the grip strength, twitch force, and tetanic force were assessed at month 8 and month 10. At month 8, the grip strength and twitch force had no significant differences between the CTL and KD groups (Figure 1c), while the tetanic force in the KD group was higher than that of the CTL group (Figure 1c). At month 10, there was still no significant difference in grip strength between the CTL and KD groups (Figure 1c). However, the KD group exhibited higher twitch and tetanic force than the CTL group (Figure 1c). The results indicated that AAV9 multi‐point local injections impacted only the limbs rather than the whole body, leading to increased twitch and tetanic forces during the progression of sarcopenia in SAMP8 mice. We also compared the grip strength, twitch force, and tetanic force between KD and KD‐NC groups, while the results showed no significant differences at both month 8 and 10 (Figure S2d). Meanwhile, we found that grip strength and twitch force had no significant differences between the KD and KD‐NC groups, while the tetanic force in the KD group was higher than that of the KD‐NC group (Figure S2d).

### 3.3 LMHFV Treatment Improved Muscle Performance by Inhibiting miR‐378

To investigate if LMHFV had effects on miR‐378 and muscle performance, we conducted LMHFV treatment for mice when they were 6‐month‐old (Figure 1d). The results illustrated that VIB group had significantly lower miR‐378 expression than the CTL group (Figure 1b). Interestingly, there was no significant difference between the VIB and KD groups (Figure 1b). These results suggested that LMHFV inhibited miR‐378 expression.

Meanwhile, we evaluated the grip strength, twitch and tetanic forces after LMHFV treatment. At month 8, there was no notable difference in grip strength between the VIB and CTL groups (Figure 1c), however, both twitch force and tetanic force were elevated in the VIB group compared to the CTL group (Figure 1c). At month 10, a significant increase in grip strength was observed in the VIB group over the CTL group (Figure 1c), with elevation in twitch force and tetanic force (Figure 1c). Interestingly, the VIB group had higher twitch force than the KD group at month 10 (Figure 1c), indicating that LMHFV improved twitch force better than miR‐378 knockdown. Moreover, the data elucidated that LMHFV enhanced twitch force more effectively when mice were in sarcopenia status.

Besides using the SAMP8 mice model, we also evaluated the muscle performance in over‐expressing miR‐378 transgenic mice. Our data showed that TG mice had lower body weight than WT mice at the age of 8 months (Figure S2e). Additionally, the weight of GA muscle normalized by body weight showed no difference between WT and TG mice (Figure S2e). The WT group had higher grip strength than the TG group (Figure 1e), possibly due to the global miR‐378 over‐expression. Similarly, WT group had higher twitch and tetanic force than the TG group (Figure 1e). Moreover, we found that over‐expressing miR‐378 reduced grip strength, twitch force, and tetanic force, while LMHFV significantly improved muscle performance in the T + V group compared to the TG group (Figure 1e). These results indicated that miR‐378 over‐expression impaired muscle strength. Moreover, it was conceivable that LMHFV improved muscle performance by inhibiting miR‐378.

### 3.4 LMHFV Attenuated Muscle Atrophy by Inhibiting miR‐378

Then, cross‐sectional area (CSA) of the GA muscle from month 3 to 10 was evaluated. The CSA exhibited an upward trend from month 3 to 8, followed by a decline from month 8 to 10 (Figure S3a), mirroring the pattern observed in grip strength, twitch force and tetanic force. These findings collectively suggested that sarcopenia onset occurs at month 8, consistent with our previous research [24].

**FIGURE 2:**
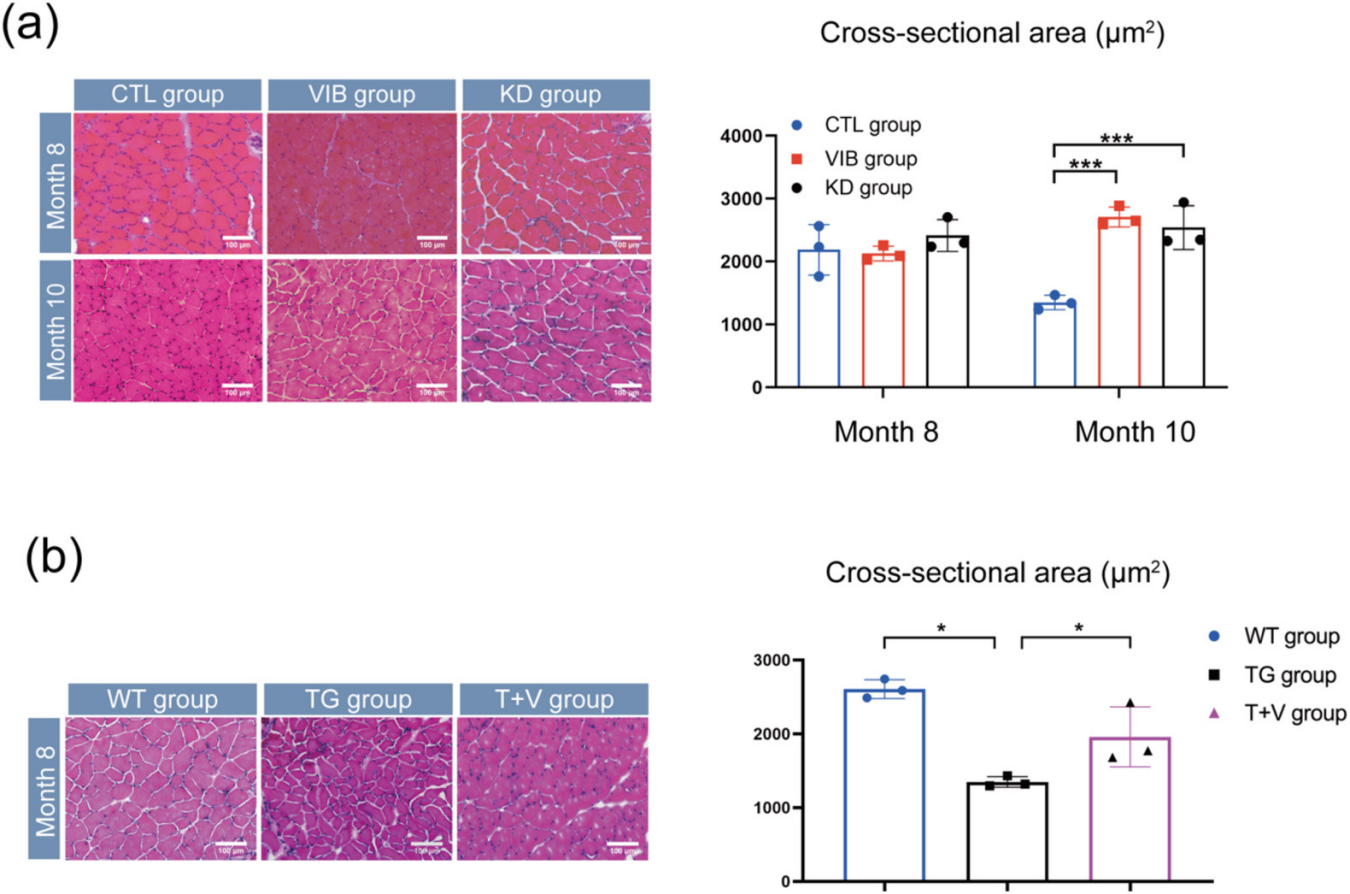
Muscle atrophy was attenuated by LMHFV treatment or knockdown miR‐378. (a) Representative H&E staining images and average cross‐sectional area of GA muscle from SAMP8 mice at month 8 and 10. Scale bar: 100 μm. (b) Representative H&E staining images and average cross‐sectional area of GA muscle from wide‐type mice and transgenic mice at month 8. Scale bar: 100 μm. Means ± SD are shown, *N* = 3, *p < 0.05, ***p < 0.006, one‐way ANOVA.

We then assessed the CSA of GA muscle after AAV9 injection. The results showed no significant differences in the CSA of GA muscle between CTL and KD groups at month 8 (Figure 2a). However, at month 10, the KD group had a larger CSA of GA muscle than that of the CTL group (Figure 2a). These results suggested that miR‐378 knockdown would attenuate muscle atrophy during progression of sarcopenia in SAMP8 mice. Furthermore, to investigate whether LMHFV could attenuate muscle fibre atrophy, H&E staining of GA muscle in the VIB group was executed. Although there were no significant changes in CSA of GA muscle at month 8, an increase was noted after LMHFV treatment at month 10 (Figure 2a).

In contrast, we observed that the CSA in the TG group was smaller than that of the WT group (Figure 2b), which suggested that miR‐378 over‐expression would exacerbate muscle atrophy. Moreover, we found that LMHFV increased the CSA of GA muscle but not significantly compared to the WT group (Figure 2b).

### 3.5 LMHFV Attenuated Mitochondrial Morphological Deterioration via Inhibiting miR‐378

We investigated mitochondrial morphological changes in GA muscle from month 3 to 10 in SAMP8 mice model. Notably, the results demonstrated a decrease in mitochondrial number, density, and relative area from month 6 to 10 (Figure S3b and c). This trend was inconsistent with miR‐378 level during the progression of sarcopenia in SAMP8 mice (Figure 1a). These observations highlight the role of mitochondrial morphological deterioration in the progression of sarcopenia in SAMP8 mice. Additionally, the data indicated that mir‐378 could damage mitochondria during progression of sarcopenia.

**FIGURE 3:**
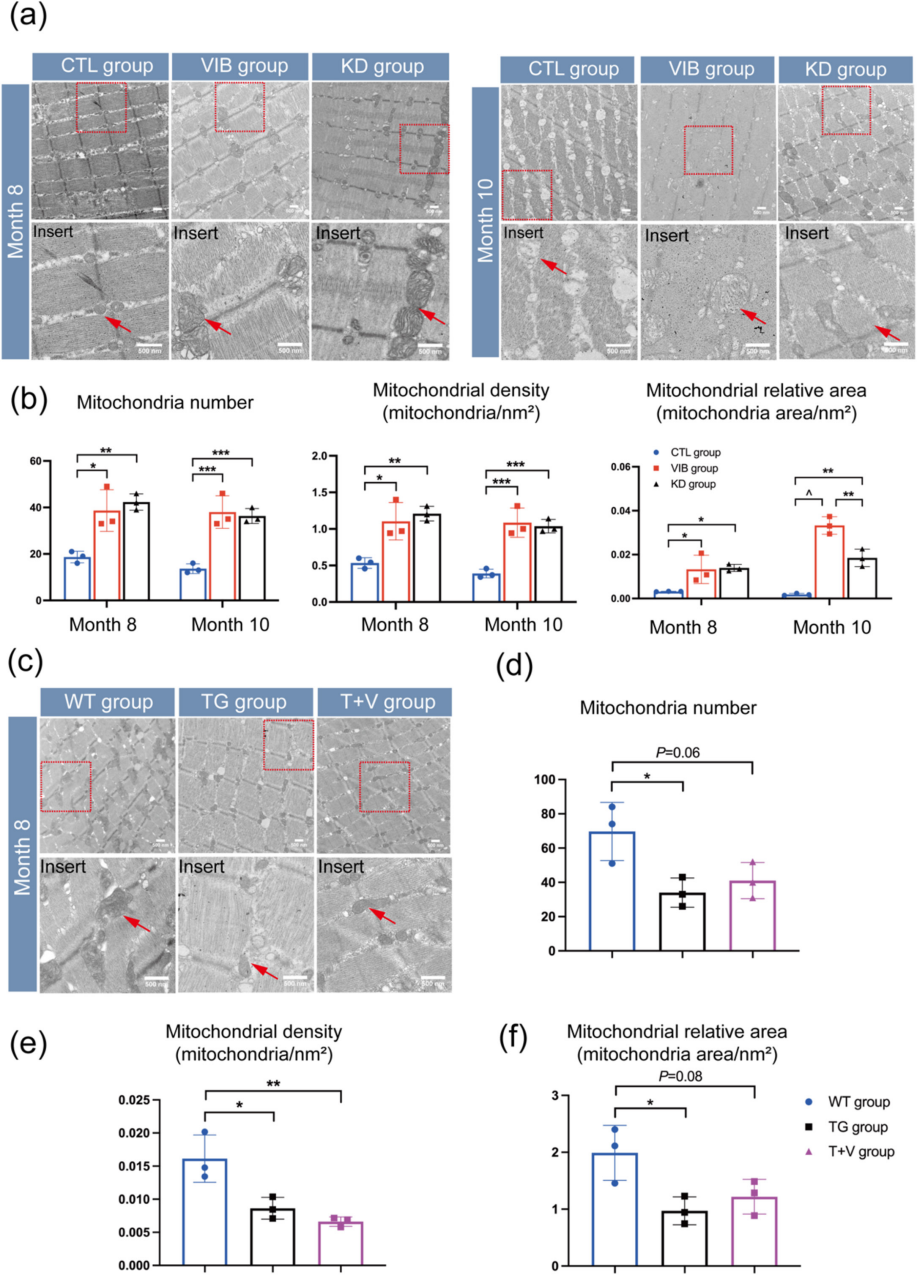
Mitochondrial morphological deterioration was attenuated by LMHFV treatment or knockdown miR‐378. (a), (b) Representative TEM images of mitochondria and quantification of mitochondrial number, density, and relative area from GA muscle in SAMP8 mice at month 8 and 10. (c) to (f) Representative TEM images of mitochondria of GA muscle in wide‐type mice and transgenic mice at month 8. Arrows indicate the mitochondria with normal cristae. Scale bar: 500 nm. Means ± SD are shown, *N* = 3, *p < 0.05, **p < 0.04, ***p < 0.006, ^*p* < 0.0001, one‐way ANOVA.

Subsequently, we assessed mitochondrial morphologies and functions after miR‐378 knockdown. The results showed that miR‐378 knockdown increased the mitochondrial number, density, and relative area in SAMP8 mice at month 8 and 10 (Figure 3a,b). Furthermore, to evaluate the effects of LMHFV on mitochondrial morphology, we compared mitochondrial number, density, and relative area between the CTL and VIB groups. The results showed that LMHFV improved these parameters at month 8 and 10 (Figure 3a,b).

Furthermore, we compared mitochondrial morphological changes between the WT and TG groups. We found that miR‐378 over‐expression decreased the mitochondrial number, density, and relative area (Figure 3c to f). These findings indicated that miR‐378 adversely affected mitochondrial morphologies during sarcopenia progression. Interestingly, the data showed that LMHFV did not significantly improve the mitochondrial number, density, and relative area compared to the TG group (Figure 3c to f).

### 3.6 Mitochondrial Homeostasis Was Damaged During Sarcopenia Progression

**FIGURE 4:**
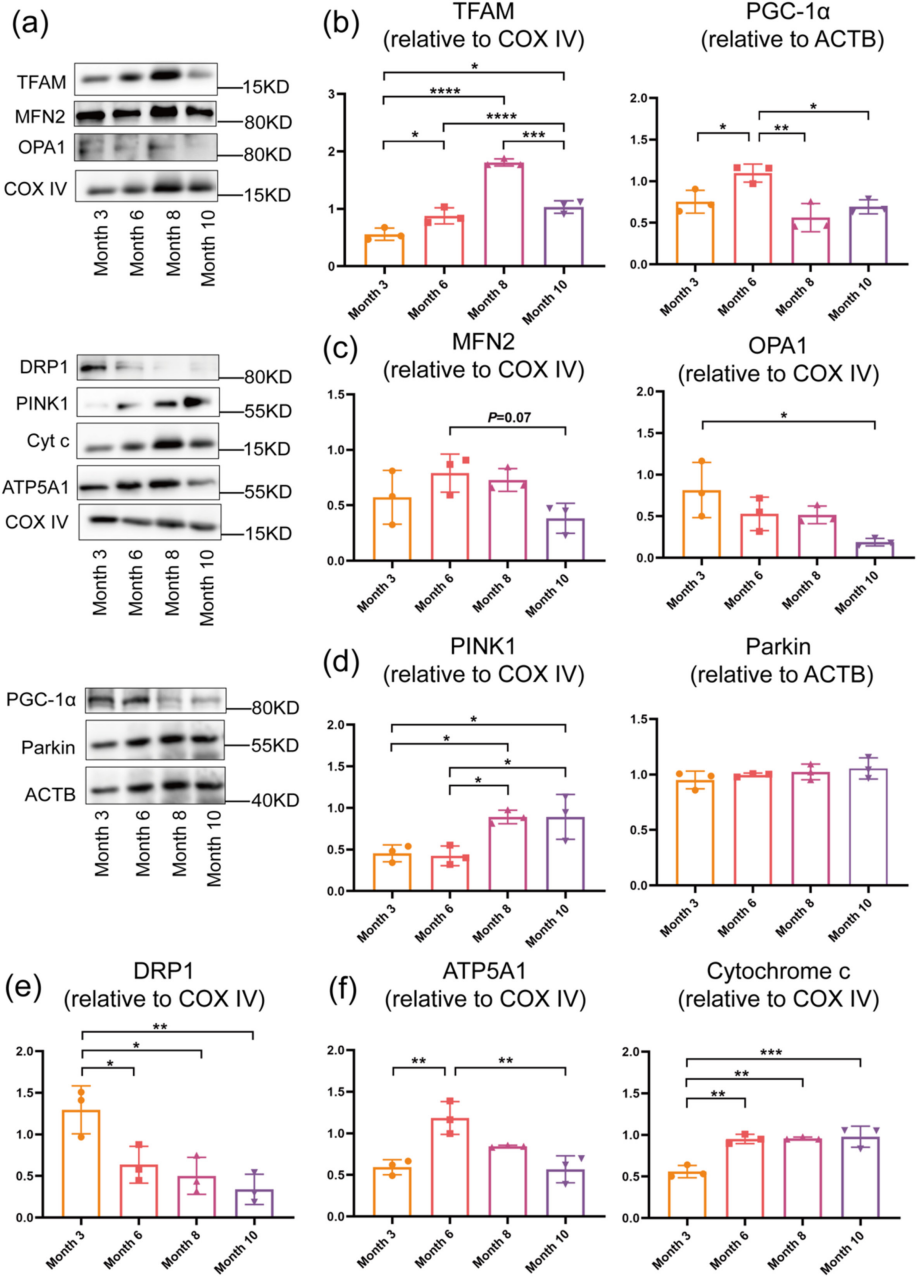
The Western blot results of mitochondrial dynamic equilibrium in SAMP8 mice from month 3 to 10. (a), (b) Representative proteins and quantification of mitochondrial biogenesis. (a), (c) Representative proteins and quantification of mitochondrial fusion. (a), (d) Representative proteins of mitophagy. (a), (e) Representative proteins of mitochondrial fission. (a), (f) Representative proteins of mitochondrial ATP production. Means ± SD are shown, *N* = 3, *p < 0.05, **p < 0.01, ***p < 0.006, one‐way ANOVA.

Mitochondrial dynamic equilibrium during sarcopenia progression in SAMP8 mice was investigated by evaluating mitochondrial biogenesis, fusion, fission, and mitophagy at protein level using cytoplasmic and mitochondrial protein from the TA muscle. To assess mitochondrial biogenesis, the levels of TFAM (a mitochondrial protein) and PGC‐1α (a cytoplasmic protein) were measured. TFAM level increased from month 3 to 8 but decreased from month 8 to 10 (Figure 4a,b). Meanwhile, PGC‐1α increased from month 3 to 6 but decreased from month 6 to 10 (Figure 4a,b). These results showed that mitochondrial biogenesis decreased no later than month 8, consistent with trends in muscle performance (Figure S2a) and muscle atrophy (Figure S3a). To assess mitochondrial fusion, the expression levels of MFN2 and OPA1 (mitochondrial proteins) were evaluated. MFN2 and OPA1 levels decreased from months 6 to 10 (Figure 4a,c), indicating reduced mitochondrial fusion during the progression of sarcopenia. This observation aligned with mitochondrial morphological deterioration (Figure S3b and c) occurring earlier than changes in muscle performance (Figure S2a) and muscle atrophy (Figure S3a). To investigate the level of mitophagy, the expression levels of PINK1 (a mitochondrial protein) and Parkin (a cytoplasmic protein) were evaluated. The level of PINK1 increased from month 3 to 10 while Parkin showed no significant changes from months 3 to 10 in SAMP8 mice (Figure 4a,d). The data suggested that the level of mitophagy decreased in SAMP8 mice model during sarcopenia progression. BINP3, LC3, and p62 are key proteins involved in mitophagy. BINP3 recruits LC3 to mitochondria to initiate mitophagy, while p62 serves as a key cargo adaptor that is degraded in the process, making its levels a proxy for autophagic activity [32]. Using TA muscle from natural ageing mice, we found that the protein level of p62 increased, whereas BNIP3 and LC3 showed no significant changes (Figure S3d). The data suggested that mitophagy declined during ageing, contributing to mitochondrial dysfunction. To investigate the level of mitochondrial fission, DRP1 expression level was evaluated. The level of DRP1 (a mitochondrial protein) decreased from month 3 to 10 (Figure 4a,e). To further explore the level of mitochondrial ATP production, the levels of cytochrome c and ATP5A1 (mitochondria proteins) were assessed. The level of cytochrome c increased from months 3 to 10, while ATP5A1 increased from months 3 to 6 but decreased from months 6 to 10 (Figure 4a,f).

### 3.7 LMHFV Treatment Attenuated Mitochondrial Homeostasis Declined by Inhibiting miR‐378

**FIGURE 5:**
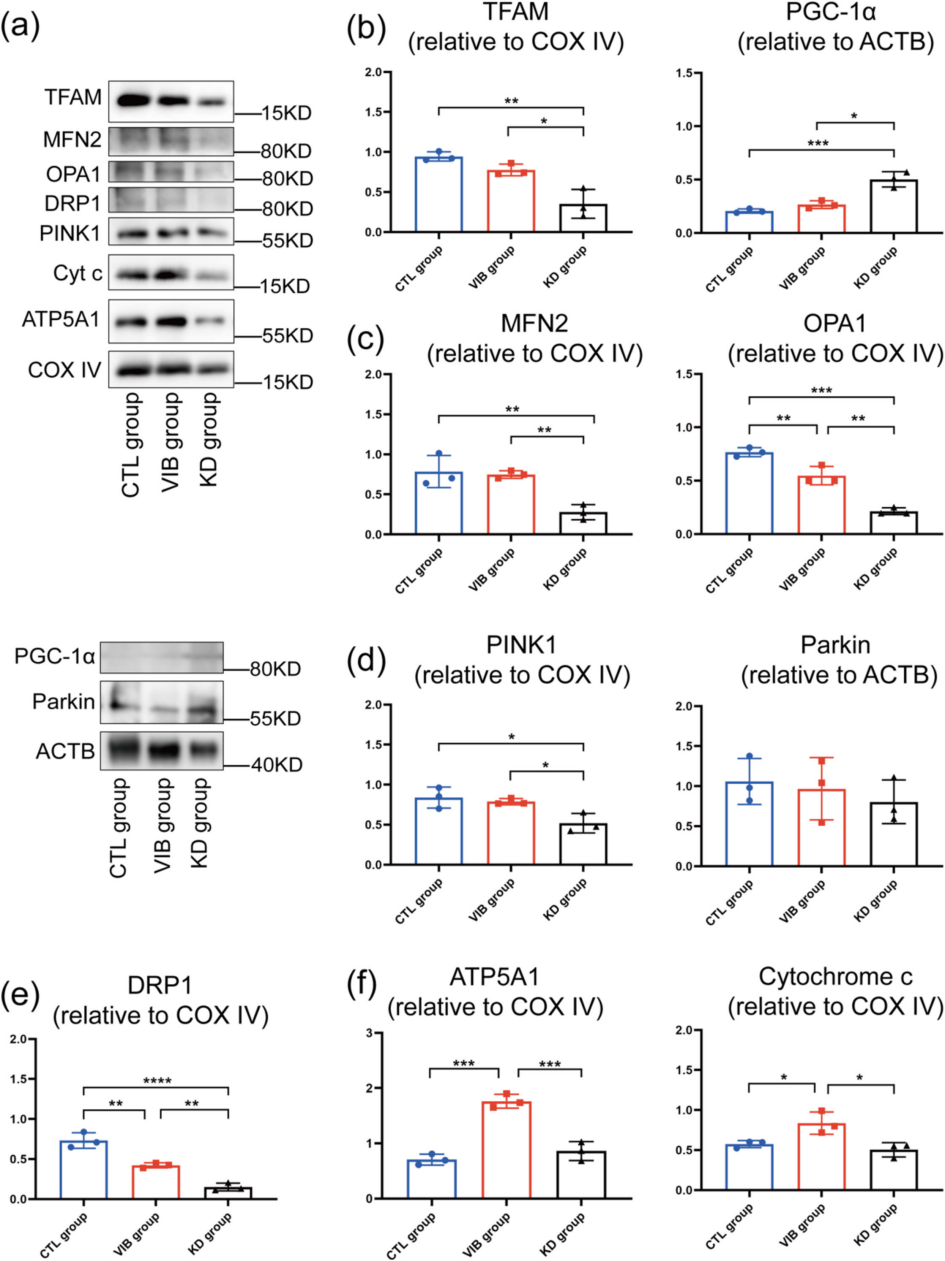
The Western blot results of mitochondrial functions of SAMP8 mice at month 10. (a), (b) Representative proteins and quantification of mitochondrial biogenesis. (a), (c) Representative proteins and quantification of mitochondrial fusion. (a), (d) Representative proteins of mitophagy. (a), (e) Representative proteins of mitochondrial fission. (a), (f) Representative proteins of mitochondrial ATP production. Means ± SD are shown, *N* = 3, *p < 0.05, **p < 0.01, ***p < 0.006, one‐way ANOVA.

Subsequently, we evaluated the effects of miR‐378 on mitochondrial quality control in SAMP8 mice at month 8 (Figure S4) and month 10 (Figure 5). For mitochondrial biogenesis, miR‐378 knockdown increased PGC‐1α (Figure S4a,b) but had no significant effect on TFAM (Figure S4a and b) at month 8, while inhibiting miR‐378 increased PGC‐1α and decreased TFAM at month 10 (Figure 5a,b). Regarding mitochondrial fusion, the KD group had lower protein level of OPA1 at month 8 (Figure S4a,c), while MFN2 and OPA1 decreased simultaneously in KD group at month 10 (Figure 3a,c). Meanwhile, DRP1 decreased in the KD group at month 8 (Figure S4a,e) and month 10 (Figure 5a,e). These data suggested that miR‐378 knockdown reduced mitochondrial fusion and fission during sarcopenia progression in SAMP8 mice. Moreover, we found a reduction of PINK1 and Parkin protein levels in the KD group at month 8 (Figure S4a,d). At month 10, however, the Parkin protein level in the KD group showed no significant changes at month 10 (Figure 5a,d). These data revealed that miR‐378 knockdown reduced mitophagy levels during sarcopenia progression in SAMP8 mice. We also assessed mitochondrial ATP production and found an increase in ATP5A1 protein levels in the KD group at month 8 (Figure S4a,f) but with no significant change at month 10 (Figure 5a,f).

To further investigate the effects of LMHFV on mitochondrial homeostasis during sarcopenia progression in SAMP8 mice, the protein levels at month 8 and 10 were assessed. At month 8, LMHFV increased the protein levels of PGC‐1α, PINK1, Parkin, cytochrome c and ATP5A1 (Figure S4a,d,f) but decreased OPA1 and DRP1 (Figure S4a, c, e). At month 10, the VIB group had similar levels of PGC‐1α, TFAM, MFN2, PINK1, and Parkin as the CTL group (Figure 5a to d). Meanwhile, LMHFV increased protein levels of cytochrome c and ATP5A1 (Figure 5d,f) but decreased OPA1 and DRP1 (Figure 5a, b, e).

**FIGURE 6:**
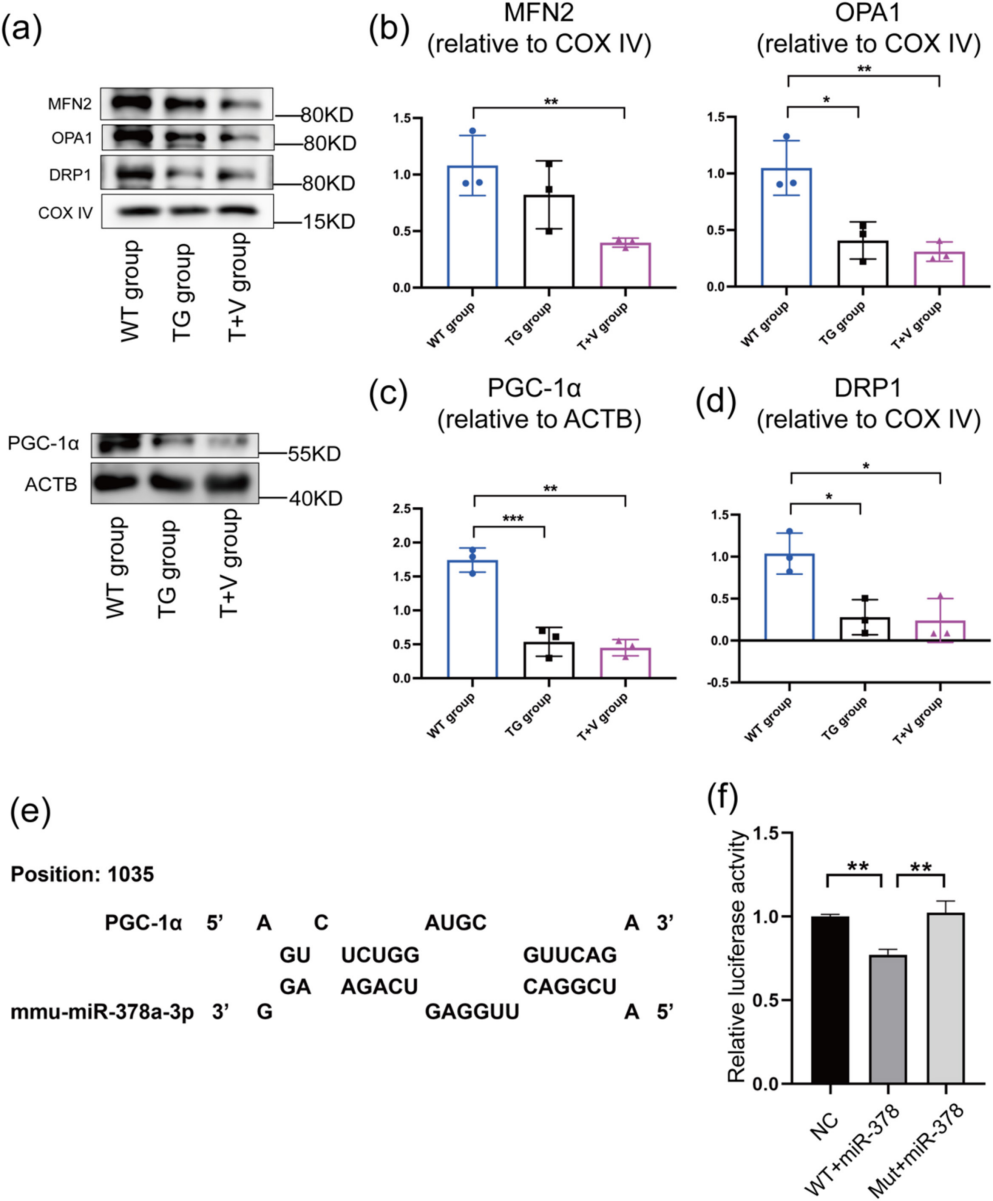
The Western blot results of mitochondrial functions of wild‐type mice, transgenic mice at month 8. (a), (b) Representative proteins and quantification of mitochondrial fusion. (a), (c) Representative proteins and quantification of mitochondrial biogenesis. (a), (d) Representative proteins of mitochondrial fission. (e) Sequence alignment of miR‐378 and the PGC‐1α 3’UTR from mouse. (f) Relative luciferase activity in the NC group, WT + miR‐378 group, and Mut + miR‐378 group. Means ± SD are shown, *N* = 3, *p < 0.05, **p < 0.01, ***p < 0.006.

In our study of mitochondrial homeostasis using miR‐378 over‐expressing mice aged 8 months, we noted a decline in PGC‐1α, OPA1, and DRP1 protein levels (Figure 6a to d), while PINK1 and Parkin remained unaffected (Figure S5a,c,d). Similarly, ATP5A1 level was stable in the TG group (Figure S5a,e). Additionally, the dual‐luciferase reporter assay was applied to validate the potential target gene of miR‐378, and the results suggested miR‐378 inhibited PGC‐1α directly (Figure 6e,f). These results suggested that while miR‐378 over‐expression inhibited mitochondrial biogenesis, fusion, and fission processes, it did not significantly impact mitophagy or ATP production.

**FIGURE 7:**
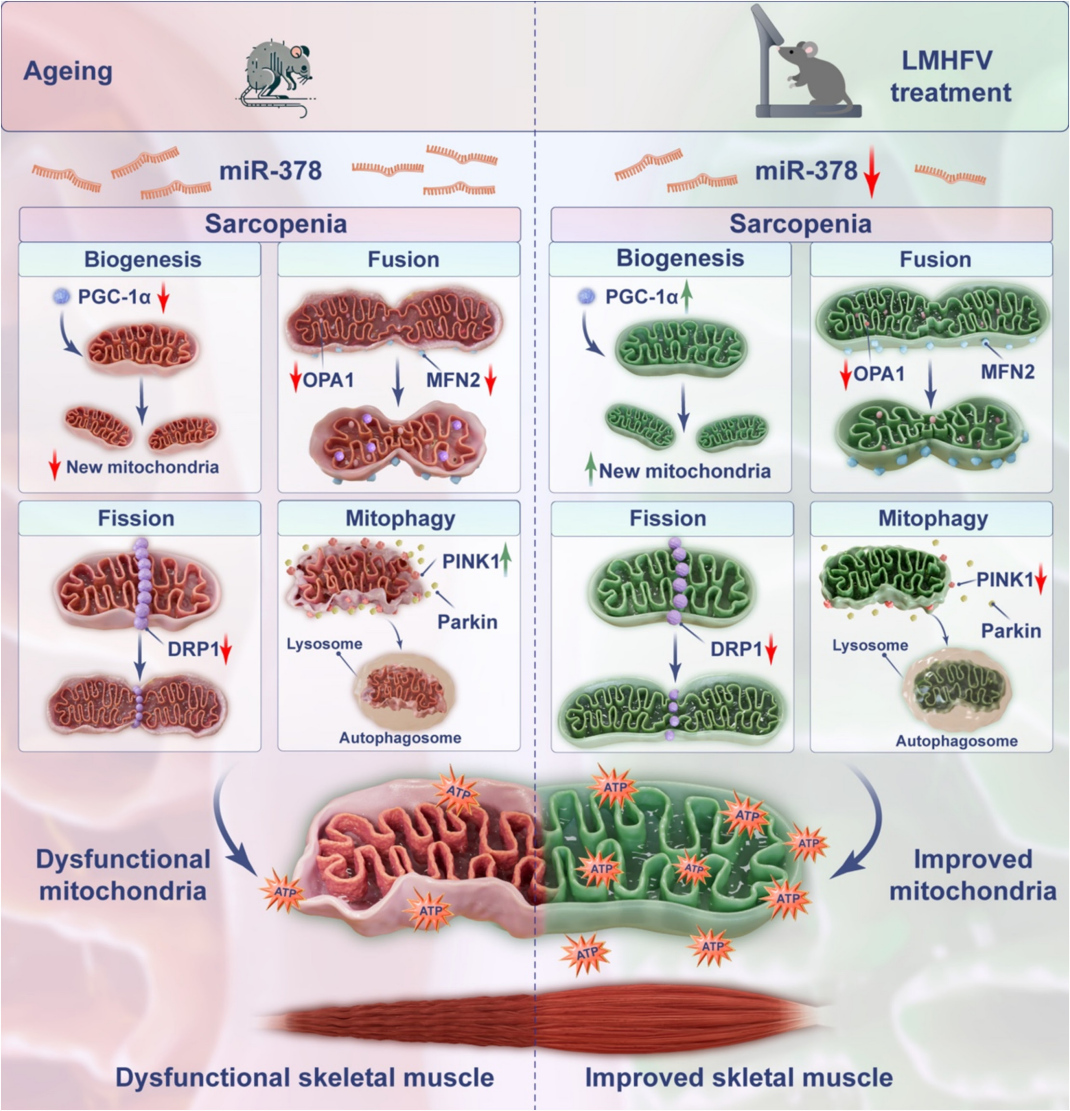
Schematic diagram of the relationships among LMHFV, miR‐378, mitochondria, and sarcopenia. LMHFV attenuated sarcopenia by improving mitochondrial function through increasing PGC‐1α via inhibiting miR‐378 in skeletal muscle.

Moreover, we examined the mitochondrial homeostasis in the T + V group to see whether LMHFV regulated it by inhibiting miR‐378. The protein levels of PGC‐1α, MFN2, OPA1, and DRP1 were lower in the T + V group than in the WT group, while there were no significant differences the TG and T + V groups (Figure 6a to d). Additionally, the proteins levels of TFAM, PINK1, Parkin, ATP5A1, and cytochrome c showed no significant changes (Figure S5). These results suggested that over‐expressing miR‐378 restricted the effects of LMHFV on improving the mitochondrial number, density, and relative area, as well as mitochondrial homeostasis. Taken together, our results illustrated that LMHFV could attenuate sarcopenia through modulating mitochondrial homeostasis by inhibiting miR‐378 in skeletal muscle (Figure 7).

## 4 Discussion

With the increasing prevalence of sarcopenia [2, 33], a better understanding of the mechanism of sarcopenia may help us develop more effective therapies to attenuate sarcopenia for the elderly population. However, the underlying relationship between mitochondria and sarcopenia has not been completely understood [34]. For instance, it was unclear if sarcopenia was the consequence or cause of mitochondrial dysfunction [7]. Here we showed that mitochondrial dysfunction preceded sarcopenia and could be the cause of sarcopenia. Meanwhile, increasing miR‐378 resulted in damaging mitochondrial homeostasis via inhibiting PGC‐1α during sarcopenia progression. Furthermore, we first elucidated that LMHFV alleviated sarcopenia by enhancing mitochondrial homeostasis through miR‐378 inhibition.

Growing evidence suggested that mitochondrial dysfunction was associated with the development of many diseases including musculoskeletal system disorder [5, 6, 35]. Using SAMP8 mice model, a proven suitable animal model for studying sarcopenia [24, 29, 36], we observed that mitochondrial number, density, and relative area decreased and were consistent with the level of mitochondrial quality control at protein level showed by western blotting. There is a consensus on the assumption that mitochondrial dysfunction is the main factor leading to muscle degeneration [37, 38]. Our data revealed that mitochondrial deterioration in skeletal muscle occurred earlier than muscle atrophy, which contributed to sarcopenia during ageing. Although microRNAs regulated muscle function via modulating mitochondria, the mechanisms of miRNAs in sarcopenia were not clear [10]. While it has been reported that miR‐378 regulated muscle atrophy and mitochondria [12, 39], our study first revealed that miR‐378 might impair muscle and mitochondria during sarcopenia progression. Meanwhile, we elucidated that miR‐378 knockdown improved muscle CSA and muscle strength, as well as attenuated mitochondrial morphological deterioration during sarcopenia progression.

Mitochondrial biogenesis is a key process for generating mitochondria, while mitochondrial fission and mitophagy are important for eliminating damaged mitochondria [6, 40]. MiRNAs showed effects on regulating mitochondria [10], and it has been reported that miR‐378 has a crucial role in the regulation of mitochondrial metabolisms [12], S1. Our data revealed that miR‐378 knockdown improved mitochondrial biogenesis but decreased mitochondrial fusion, fission, and mitophagy. Moreover, knocking down miR‐378 improved mitochondrial biogenesis and decreased mitochondrial fission and mitophagy could explain the increased mitochondrial number, density, and relative area in the KD group. Adenosine triphosphate (ATP) is a crucial molecule for powering the biochemical reactions for life. Previous studies indicated that miR‐378 could enhance lipolysis [26], decrease ATP synthase protein content [S2], and damage energy homeostasis [39]. Interestingly, our data revealed that miR‐378 knockdown increased mitochondrial ATP production level at month 8 but showed no significant effect at month 10. According to our previous studies [27, 28], SAMP8 mice were ageing from month 8 to 10, which means lower anabolic level during this period. This may be one of reasons why miR‐378 knockdown showed no significant effect on improving mitochondrial ATP production level.

MicroRNAs participated in a regulatory circuit that allowed rapid gene program transitions from proliferation to differentiation in muscle [S3]. It has been elucidated that miR‐378 caused muscle atrophy by blocking apoptosis and enhancing autophagy in skeletal muscle [12]. Our data also revealed that mitophagy declined during ageing in skeletal muscle, contributing to muscle atrophy. Although over‐expressing miR‐378 mice has been used in musculoskeletal system‐related studies [12, S4], few studies used the transgenic mice to explore the role of miR‐378 in sarcopenia and mitochondria. Herein, we used TG mice to investigate the effects of miR‐378 on muscle and mitochondria for having comprehensive understanding of miR‐378 in skeletal muscle. The data revealed that miR‐378 over‐expressing decreased muscle CSA and muscle strength simultaneously. Meanwhile, the results elucidated that miR‐378 over‐expression decreased mitochondrial biogenesis, fusion, and fission significantly in skeletal muscle. Based on the data from SAMP8 mice and transgenic mice, it was conceivable that miR‐378 could cause sarcopenia by damaging mitochondrial homeostasis. Moreover, our data elucidated that miR‐378 played a key role in sarcopenia progression by impairing mitochondrial biogenesis via inhibiting PGC‐1α.

LMHFV treatment is a kind of intriguing intervention that uses oscillation to provide mechanical transfer to the whole body through minimal vertical movement [29]. CDC has recommended using LMHFV treatment to reduce risk of falls among elderly [25]. In this study, knockdown miR‐378 had no effect on improving grip strength but LMHFV increased grip strength significantly. Since knockdown miR‐378 was performed by muscle local multi‐point injection, this could be the reason why the grip strength in KD group and CTL group had no difference. Meanwhile, the data proved that LMHFV was a treatment to the whole body. Although our previous studies revealed that LMHFV treatment attenuated sarcopenia, the underlying molecular mechanism of this was undiscovered [24, 29]. A randomized controlled trial in postmenopausal women showed that vibration treatment had effects on regulating miRNAs, while resistance exercise did not influence miRNAs significantly [S5]. Herein, our data first illustrated that LMHFV could attenuate sarcopenia via modulating mitochondria by inhibiting miR‐378 in skeletal muscle.

It is well‐established that mitochondria are the powerhouse of the cell due to their central role in energy production, and the primary function of mitochondria is to produce energy for the cell and maintain cellular metabolism [S6]. Interestingly, the whole‐body vibration treatment could improve mitochondrial homeostasis in skeletal muscle [S7, S8]. Our data that LMHFV improved mitochondrial biogenesis, mitophagy, and ATP production level at month 8, while the treatment showed significant effects on inhibiting mitochondrial fission and improving ATP production level at month 10. These data explain why mitochondrial number, density, and relative area in the VIB group were higher than that of the CTL group. Additionally, since mitochondrial fission is a process for improving mitochondrial number as well [S9], the inhibited mitochondrial fission and no significant change of mitophagy in SAMP8 mice at month 10 could be the reasons why LMHFV had better effect on increasing mitochondrial relative area than that of other groups.

It has been recognized that sarcopenia is characterized by decreased proportion of muscle fibre II in mice model [S10]. Our previous studies showed that muscle fibre type II decreased but LMHFV treatment increased the proportion during sarcopenia progression [27, 29]. This elucidated that LMHFV treatment attenuated sarcopenia via increasing the proportion of fast‐twitch muscle fibre. In this study, we revealed that LMHFV attenuated GA muscle fibre muscle atrophy caused by over‐expressing miR‐378. Using SAMP8 mice model, the data showed that sarcopenia was accompanied by increased miR‐378 and impaired mitochondrial homeostasis. Knocking down miR‐378 improved muscle performance and mitochondrial homeostasis. Additionally, our results suggested that LMHFV improved muscle performance and mitochondrial homeostasis, while inhibiting miR‐378. Taken together, these data revealed that LMHFV could attenuate sarcopenia. To have more comprehensive and solid evidence, we used whole‐body miR‐378 over‐expressing mice. The results showed that muscle atrophy occurred alongside impaired mitochondrial homeostasis. The data further supported that miR‐378 induced sarcopenia by damaging mitochondrial homeostasis. Since TG mice can consistently over‐express miR‐378 throughout the body [26], this explains why grip strength, twitch and tetanic forces showed no significant difference between WT and T + V groups. Furthermore, this could cause serious mitochondrial damage and muscle atrophy. In this situation, LMHFV may not show significant effects on inhibiting miR‐378, improving mitochondrial homeostasis and muscle performance.

This study has limitations. First, we only evaluated mitochondrial homeostasis in fast‐twitch muscle, which could not provide a comprehensive evaluation of mitochondrial dynamics in all types of skeletal muscle including slow‐twitch muscle. Second, although this study demonstrated mitochondrial dysfunction during sarcopenia progression, the anabolic abilities of the animal model were not examined. Third, the sample size in some experiments such as mitochondrial histological analysis, may not be sufficient to support our conclusion. Meanwhile, the CSA was estimated by dividing the total cross‐sectional area by the number of fibres, a method that may introduce measurement errors due to intercellular spaces and fat infiltration. Additionally, to have a more comprehensive understanding of the relationship between mitochondria and sarcopenia, future studies should employ natural ageing animal models and investigate metabolic changes as well as mitochondrial respiration throughout ageing, preferably with adequately powered sample sizes, which could help elucidate the pathogenesis of sarcopenia with stronger evidence. Moreover, muscle‐specific miR‐378 transgenic mice could be used in the future studies to gain a more precise understanding of the underlying mechanisms, though the whole body miR‐378 over‐expressing transgenic mice were used in this study.

To our knowledge, the mechanism of LMHFV treatment attenuates sarcopenia via modulating mitochondrial homeostasis has not been previously described. Our study revealed that mitochondrial quality control was disturbed during ageing, which could be the cause of sarcopenia. Furthermore, increased miR‐378 played an important role in impairing mitochondrial homeostasis during sarcopenia progression, especially in damaging mitochondrial biogenesis via inhibiting PGC‐1α. Moreover, our study elucidated that LMHFV attenuated sarcopenia by modulating mitochondrial quality control via inhibiting miR‐378. We elucidated that targeting miR‐378 in skeletal muscle could be a possible treatment for patients with sarcopenia. Although this is probably not the only mechanism of LMHFV treating sarcopenia, our findings imply miR‐378 could be a potential target for further investigating in clinical trials. In conclusion, this study provided new insight into mechanisms of LMHFV treating sarcopenia and inspired the potential application of mitochondrial therapy for patients with sarcopenia and limited mobility.

## Conflicts of Interest

The authors declare no conflicts of interest.

## Supporting information


**Figure S1.** (a) LMHFV treatment for SAMP8 mice or TG mice in specific pathogen‐free (SPF) animal house. The mice were put into compartment in the mice cage. (b) AAV9 multi‐point local injections in SAMP8 mice of both legs. (c) Equipment for *ex‐vivo* muscle functional test.
**Figure S2.** (a) The grip strength, twitch force, and tetanic force SAMP8 mice during progression of sarcopenia. (b) The miR‐378 expression level in EDL and SOL muscles from wide‐type mice. (c) Immunofluorescence staining of GA muscle of SMAP8 mice in the KD group. (d) The grip strength, twitch force, and tetanic force from month 8 to 10 in the CTL and KD‐NC group. *N* = 3, *p < 0.05, **p < 0.01, ***p < 0.006, ^*p* < 0.0001, one‐way ANOVA. (e) The body weight and ratio of GA muscle to body weight from WT and TG groups at month 8. Means ± SD are shown, *N* = 4–5, *p < 0.05, *t*‐test.
**Figure S3** (a) Representative H&E staining images and average cross‐sectional area of GA muscle from SAMP8 mice from month 8 to month 10. Scale bar: 100 μm. (b, c) Representative TEM images and quantification of mitochondrial number, density, and relative area of GA muscle from SAMP8 mice from month 8 to month 10; arrows indicate mitochondria. Scale bar: 500 nm. Means ± SD are shown, *N* = 3–6, *p < 0.05, **p < 0.04, ***p < 0.006, one‐way ANOVA. (d) The Western blot results of p62, BNIP3, LC3A/B of wide‐type mice.
**Figure S4.** The Western blot results of mitochondrial functions of SAMP8 mice at month 8. Means ± SD are shown, *N* = 3, *p < 0.05, **p < 0.01, ***p < 0.006, one‐way ANOVA.
**Figure S5.** The Western blot results of mitochondrial functions of WT mice and TG mice at month 8. Means ± SD are shown, *N* = 3, one‐way ANOVA.
**Table S1**. Antibodies for western blot.
**Table S2**. Information of miR‐378.
