# Supplementary material for: Low‐Magnitude High‐Frequency Vibration Attenuates Sarcopenia by Modulating Mitochondrial Quality Control via Inhibiting miR‐378
Source: J Cachexia Sarcopenia Muscle. 2025 Feb 19;16(1):e13740. doi: 10.1002/jcsm.13740 (PMC11839240; doi:10.1002/jcsm.13740)
Supplement: Supplementary file 1 — Figure S1. (a) LMHFV treatment for SAMP8 mice or TG mice in specific pathogen‐free (SPF) animal house. The mice were put into compartment in the mice cage. (b) AAV9 multi‐point local injections in SAMP8 mice of both legs. (c) Equipment for ex‐vivo muscle functional test. Figure S2. (a) The grip strength, twitch force, and tetanic force SAMP8 mice during progression of sarcopenia. (b) The miR‐378 expression level in EDL and SOL muscles from wide‐type mice. (c) Immunofluorescence staining of GA muscle of SMAP8 mice in the KD group. (d) The grip strength, twitch force, and tetanic force from month 8 to 10 in the CTL and KD‐NC group. N = 3, *p < 0.05, **p < 0.01, ***p < 0.006, ^p < 0.0001, one‐way ANOVA. (e) The body weight and ratio of GA muscle to body weight from WT and TG groups at month 8. Means ± SD are shown, N = 4–5, *p < 0.05, t‐test. Figure S3 (a) Representative H&E staining images and average cross‐sectional area of GA muscle from SAMP8 mice from month 8 to month 10. Scale bar: 100 μm. (b, c) Representative TEM images and quantification of mitochondrial number, density, and relative area of GA muscle from SAMP8 mice from month 8 to month 10; arrows indicate mitochondria. Scale bar: 500 nm. Means ± SD are shown, N = 3–6, *p < 0.05, **p < 0.04, ***p < 0.006, one‐way ANOVA. (d) The Western blot results of p62, BNIP3, LC3A/B of wide‐type mice. Figure S4. The Western blot results of mitochondrial functions of SAMP8 mice at month 8. Means ± SD are shown, N = 3, *p < 0.05, **p < 0.01, ***p < 0.006, one‐way ANOVA. Figure S5. The Western blot results of mitochondrial functions of WT mice and TG mice at month 8. Means ± SD are shown, N = 3, one‐way ANOVA. Table S1. Antibodies for western blot. Table S2. Information of miR‐378. [file JCSM-16-e13740-s001.docx]

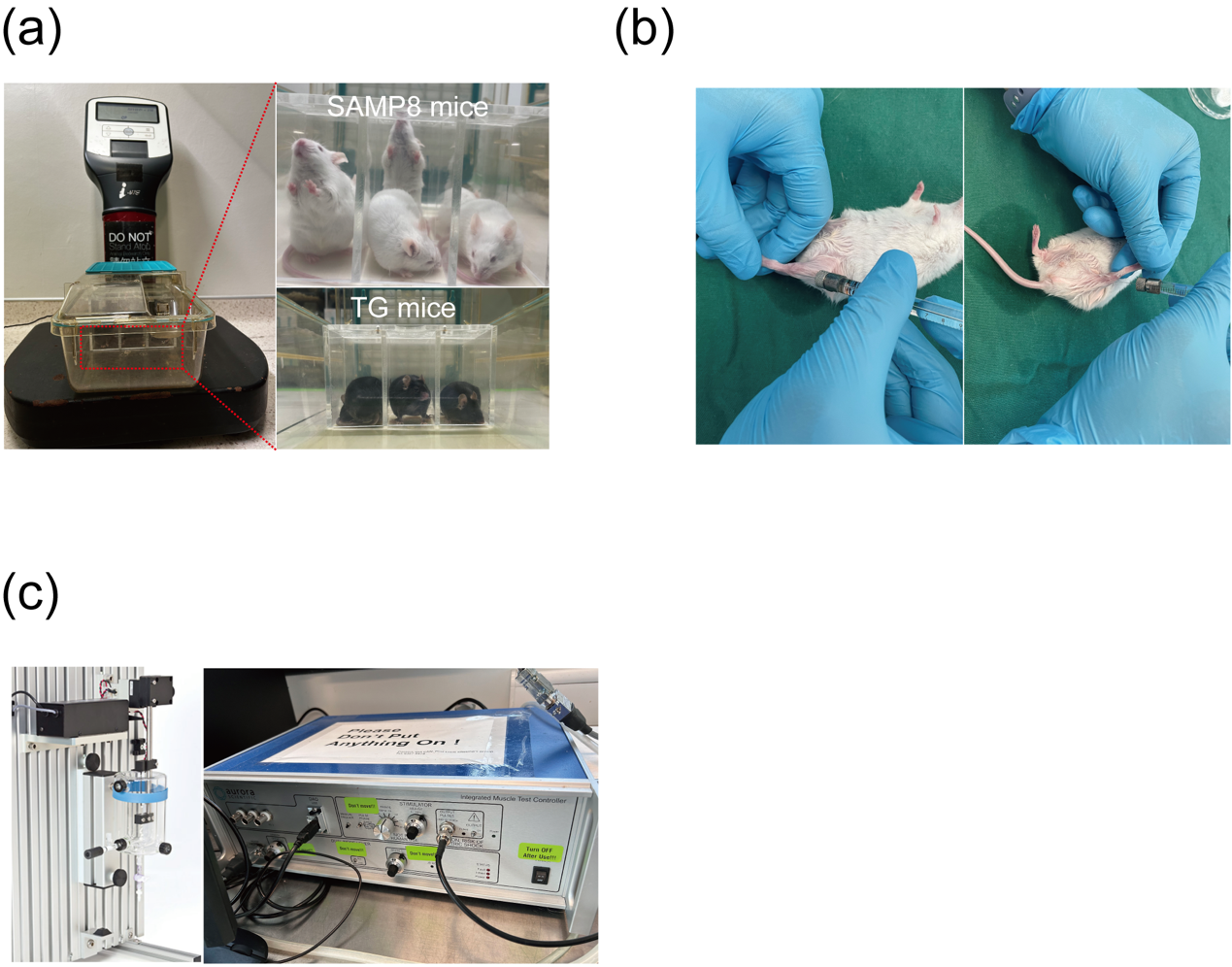


**Figure S1** (a) LMHFV treatment for SAMP8 mice or TG mice in specific pathogen-free (SPF) animal house. The mice were put into compartment in the mice cage. (b) AAV9 muti-point local injections in SAMP8 mice of both legs. (c) Equipment for *ex-vivo* muscle functional test.

**
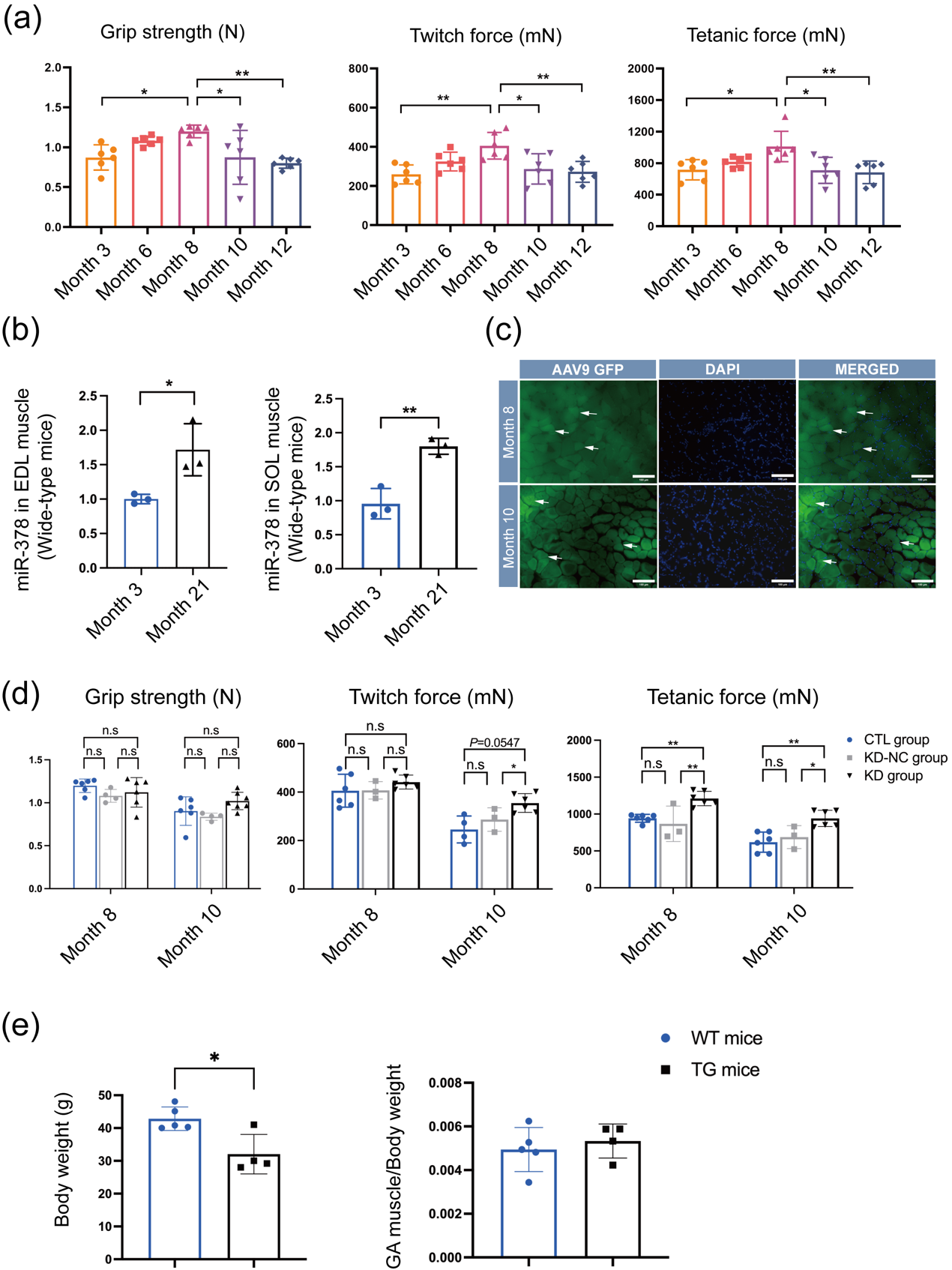
 Figure S2** (a) The grip strength, twitch force, and tetanic force SAMP8 mice during progression of sarcopenia. (b) The miR-378 expression level in EDL and SOL muscles from wide-type mice. (c) Immunofluorescence staining of GA muscle of SMAP8 mice in the KD group. (d) The grip strength, twitch force, and tetanic force from month 8 to 10 in the CTL and KD-NC group. *N*=3, **P* <0.05, ***P*<0.01, ****P*<0.006, ^*P*<0.0001, one-way ANOVA. (e) The body weight and ratio of GA muscle to body weight from WT and TG groups at month 8. Means ± SD are shown, *N*=4-5, **P* <0.05, t-test.


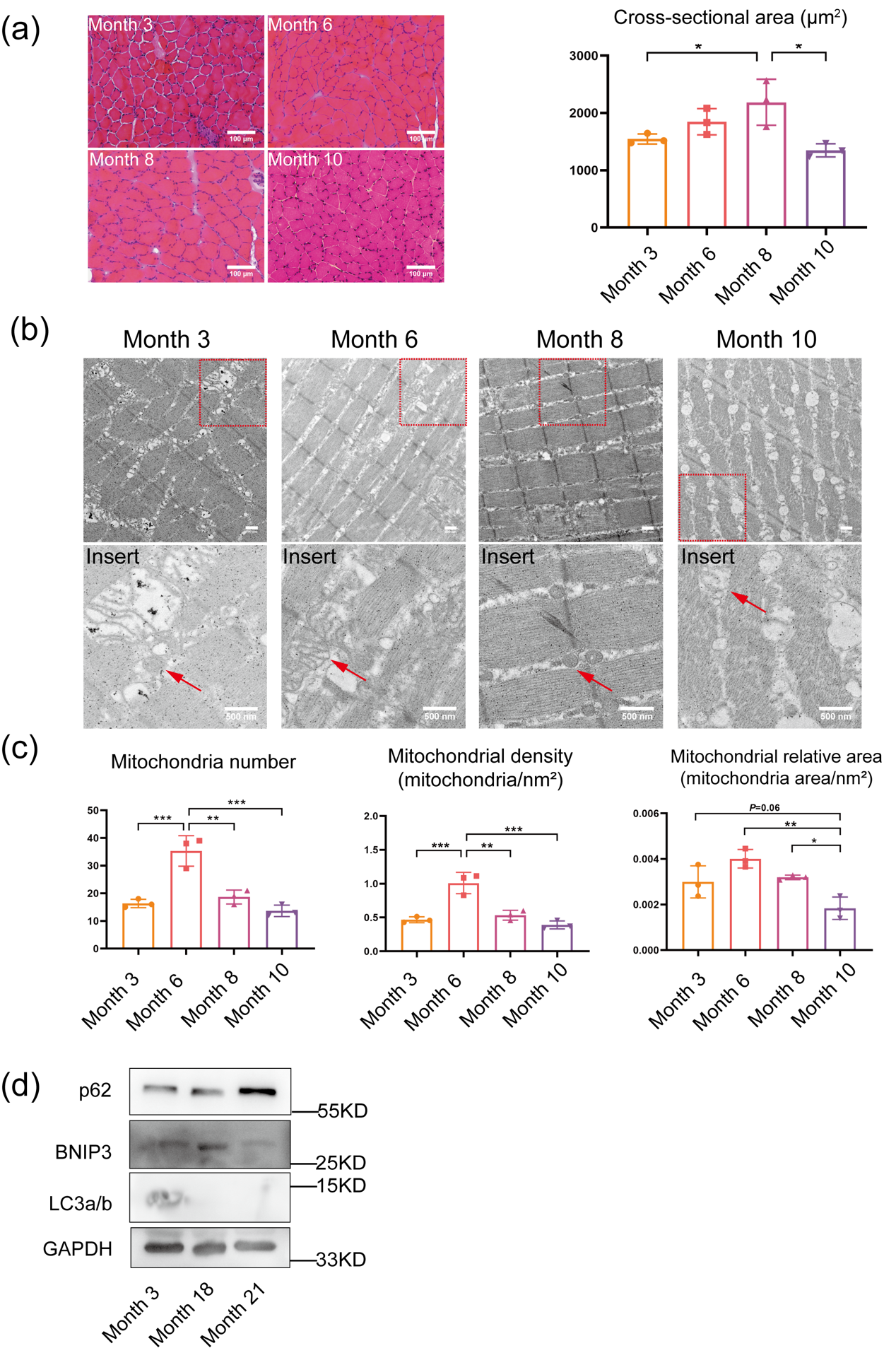


**Figure S3** (a) Representative H&E staining images and average cross-sectional area of GA muscle from SAMP8 mice from month 8 to month 10. Scale bar: 100 μm. (b, c) Representative TEM images and quantification of mitochondrial number, density, and relative area of GA muscle from SAMP8 mice from month 8 to month 10; arrows indicate mitochondria. Scale bar: 500 nm. Means ± SD are shown, *N*=3-6, **P* <0.05, ***P*<0.04, ****P*<0.006, one-way ANOVA. (d) The Western blot results of p62, BNIP3, LC3A/B of wide-type mice.


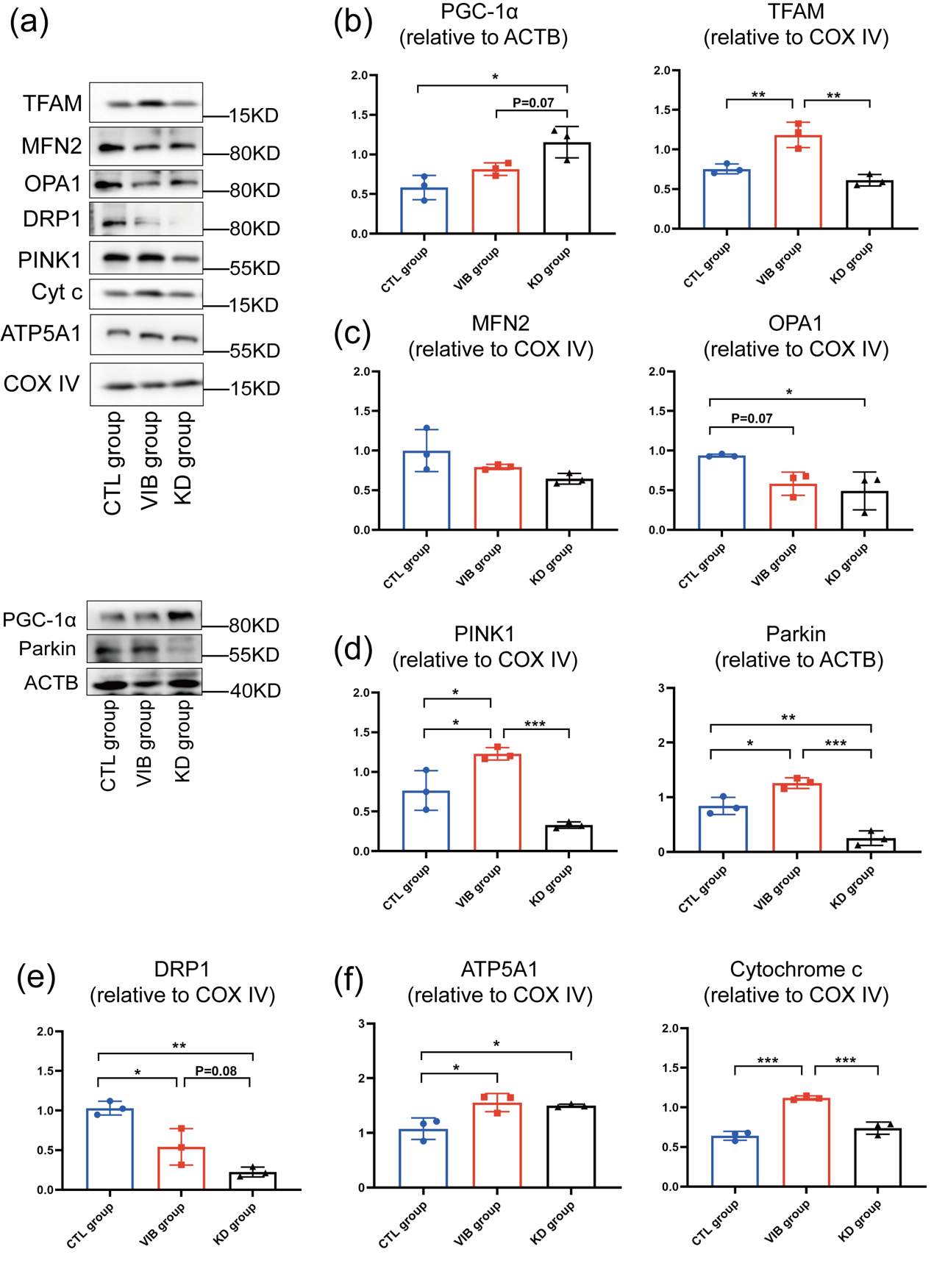


**Figure S4** The Western blot results of mitochondrial functions of SAMP8 mice at month 8. Means ± SD are shown, *N*=3, **P*<0.05, ***P*<0.01, ****P*<0.006, one-way ANOVA.


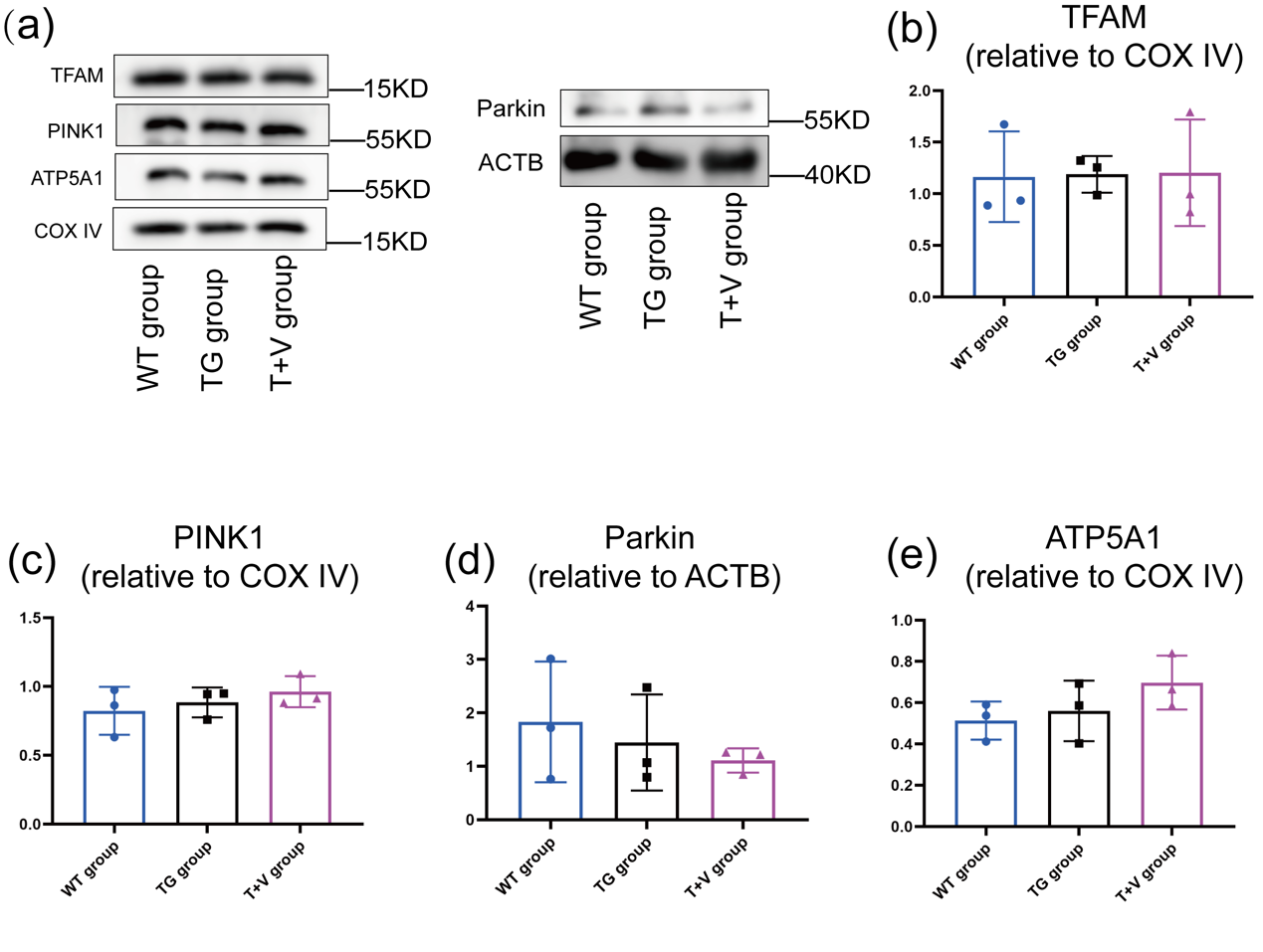


**Figure S5** The Western blot results of mitochondrial functions of WT mice and TG mice at month 8. Means ± SD are shown, *N*=3, one-way ANOVA.

**Table S1. Antibodies for western blot**

| **Antibody** | **Information** | **Dilution** |
| --- | --- | --- |
| β-Actin (ACTB) | #AC026, Abclonal | 1: 3000 |
| GAPDH | #ab128915, Abcam | 1:3000 |
| PGC-1a | #A11971, Abclonal | 1: 3000 |
| TFAM | #A1926, Abclonal | 1: 3000 |
| COX IV | #A6564, Abclonal | 1: 3000 |
| MFN2 | #ab124773, Abcam | 1: 3000 |
| OPA1 | #A9833, Abclonal | 1: 3000 |
| DRP1 | #A17069, Abclonal | 1: 3000 |
| PINK1 | #A7131, Abclonal | 1: 3000 |
| Parkin | #A0968, Abclonal | 1: 3000 |
| BNIP3 | #ab10433, Abcam | 1:3000 |
| LC3A/B | #A5618, Abclonal | 1:3000 |
| p62 | #ab109012, Abcam | 1:3000 |
| anti-rabbit IgG | #7074, Cell Signal Technology | 1:10000 |

**Table S2. Information of miR-378**

| **Iterm** | **Information** |
| --- | --- |
| miRBase ID | MIMAT0003151 |
| Sequence | ACUGGACUUGGAGUCAGAAGG |
| Viral vector | pAAV-U6-TuD (mmu-miR-378a-3p)-CMV-EGFP-WPRE |
| Non-function viral vector | pAAV-U6-shRNA (NC2)-CMV-EGFP-WPRE |

**Additional references**

S1. Machado IF, Teodoro JS, Palmeira CM, Rolo AP. miR-378a: a new emerging microRNA in metabolism. Cell Mol Life Sci. 2020;77:1947-58.

S2. Durr AJ, Hathaway QA, Kunovac A, Taylor AD, Pinti MV, Rizwan S, et al. Manipulation of the miR-378a/mt-ATP6 regulatory axis rescues ATP synthase in the diabetic heart and offers a novel role for lncRNA Kcnq1ot1. Am J Physiol Cell Physiol. 2022;322:C482-c95.

S3. Chen JF, Tao Y, Li J, Deng Z, Yan Z, Xiao X, et al. microRNA-1 and microRNA-206 regulate skeletal muscle satellite cell proliferation and differentiation by repressing Pax7. J Cell Biol. 2010;190:867-79.

S4. Feng L, Yang Z, Li Y, Pan Q, Zhang X, Wu X, et al. MicroRNA-378 contributes to osteoarthritis by regulating chondrocyte autophagy and bone marrow mesenchymal stem cell chondrogenesis. Mol Ther Nucleic Acids. 2022;28:328-41.

S5. Buchanan SR, Miller RM, Nguyen M, Black CD, Kellawan JM, Bemben MG, et al. Circulating microRNA responses to acute whole-body vibration and resistance exercise in postmenopausal women. Front Endocrinol (Lausanne). 2022;13:1038371.

S6. Nunnari J, Suomalainen A. Mitochondria: in sickness and in health. Cell. 2012;148:1145-59.

S7. Kenny HC, Rudwill F, Breen L, Salanova M, Blottner D, Heise T, et al. Bed rest and resistive vibration exercise unveil novel links between skeletal muscle mitochondrial function and insulin resistance. Diabetologia. 2017;60:1491-501.

S8. Hu Y, Fang B, Tian X, Wang H, Tian X, Yu F, et al. Passive exercise is an effective alternative to HRT for restoring OVX induced mitochondrial dysfunction in skeletal muscle. Front Endocrinol (Lausanne). 2024;15:1356312.

S9. Kleele T, Rey T, Winter J, Zaganelli S, Mahecic D, Perreten Lambert H, et al. Distinct fission signatures predict mitochondrial degradation or biogenesis. Nature. 2021;593:435-9.

S10. Larsson L, Degens H, Li M, Salviati L, Lee YI, Thompson W, et al. Sarcopenia: Aging-Related Loss of Muscle Mass and Function. Physiol Rev. 2019;99:427-511.
